# Construction of high-density linkage maps for mapping quantitative trait loci for multiple traits in field pea (*Pisum sativum* L.)

**DOI:** 10.1186/s12870-018-1368-4

**Published:** 2018-08-16

**Authors:** Krishna K. Gali, Yong Liu, Anoop Sindhu, Marwan Diapari, Arun S. K. Shunmugam, Gene Arganosa, Ketema Daba, Carolyn Caron, Reddy V. B. Lachagari, Bunyamin Tar’an, Thomas D. Warkentin

**Affiliations:** 1 Crop Development Centre, Department of Plant Sciences, 51 Campus Drive, University of Saskatchewan, Saskatoon, SK Canada; 2 CHS, Inc, 220 Clement Ave., Grandin, ND 58038 USA; 3 Agriculture and Agri-Food Canada, London Research and Development centre, 1391 Sandford Street, London, ON N5V 4T3 Canada; 4 National Research Council Canada, 110 Gymnasium Place, Saskatoon, SK S7N 0W9 Canada; 5 AgriGenome Labs Pvt Ltd., BTIC, MN iHub, Genome Valley, Shamirpet, Hyderabad, 500 078 India

**Keywords:** Pea, *Pisum sativum* L., Quantitative trait loci

## Abstract

**Background:**

The objective of this research was to map quantitative trait loci (QTLs) of multiple traits of breeding importance in pea (*Pisum sativum* L.). Three recombinant inbred line (RIL) populations, PR-02 (Orb x CDC Striker), PR-07 (Carerra x CDC Striker) and PR-15 (1–2347-144 x CDC Meadow) were phenotyped for agronomic and seed quality traits under field conditions over multiple environments in Saskatchewan, Canada. The mapping populations were genotyped using genotyping-by-sequencing (GBS) method for simultaneous single nucleotide polymorphism (SNP) discovery and construction of high-density linkage maps.

**Results:**

After filtering for read depth, segregation distortion, and missing values, 2234, 3389 and 3541 single nucleotide polymorphism (SNP) markers identified by GBS in PR-02, PR-07 and PR-15, respectively, were used for construction of genetic linkage maps. Genetic linkage groups were assigned by anchoring to SNP markers previously positioned on these linkage maps. PR-02, PR-07 and PR-15 genetic maps represented 527, 675 and 609 non-redundant loci, and cover map distances of 951.9, 1008.8 and 914.2 cM, respectively. Based on phenotyping of the three mapping populations in multiple environments, 375 QTLs were identified for important traits including days to flowering, days to maturity, lodging resistance, Mycosphaerella blight resistance, seed weight, grain yield, acid and neutral detergent fiber concentration, seed starch concentration, seed shape, seed dimpling, and concentration of seed iron, selenium and zinc. Of all the QTLs identified, the most significant in terms of explained percentage of maximum phenotypic variance (PV_max_) and occurrence in multiple environments were the QTLs for days to flowering (PV_max_ = 47.9%), plant height (PV_max_ = 65.1%), lodging resistance (PV_max_ = 35.3%), grain yield (PV_max_ = 54.2%), seed iron concentration (PV_max_ = 27.4%), and seed zinc concentration (PV_max_ = 43.2%).

**Conclusion:**

We have identified highly significant and reproducible QTLs for several agronomic and seed quality traits of breeding importance in pea. The QTLs identified will be the basis for fine mapping candidate genes, while some of the markers linked to the highly significant QTLs are useful for immediate breeding applications.

**Electronic supplementary material:**

The online version of this article (10.1186/s12870-018-1368-4) contains supplementary material, which is available to authorized users.

## Background

Pea (*Pisum sativum* L.) is one of the most widely grown pulse crops in the world, along with common bean and chickpea. Pea seeds are highly nutritious as they contain approximately 25% protein, slowly digestible carbohydrates, and a rich array of vitamins, minerals, and phytochemicals [1]. Key pea breeding objectives include increasing resistance to biotic and abiotic stresses, as well as increasing grain yield, lodging resistance, and seed mineral concentration [2]. Breeding progress over the past two decades has led to improvement in grain yield in the order of 2% per year, as well as improvements in lodging resistance, biotic stress tolerance, seed protein concentration, and improved plant architecture, in particular with the wide adoption of the *afila* gene for the semileafless trait (reviewed by [3]).

Molecular markers including single nucleotide polymorphisms (SNPs), simple sequence repeats (SSRs) and other markers have been used to study the genetic variation within *Pisum*. These markers were useful for the construction of linkage maps to provide frameworks for identification of quantitative trait loci (QTLs) and trait-linked markers related to many important traits including resistance to diseases such as powdery mildew, Fusarium wilt, Ascochyta blight and rust, lodging resistance, time to flowering, seed mineral and protein concentration (reviewed by [2–5]), and validation of some of the identified QTLs (eg. [6]). However, the QTLs identified using these markers had large confidence intervals due to low resolution genetic maps and uneven distribution of markers. Therefore, development of high density genetic linkage maps is important for QTL identification and marker-assisted breeding.

SNP markers have a wide occurrence across the genome, thus are an ideal choice for construction of high density linkage maps and identification of markers closely linked to traits [7]. With the use of next generation sequencing, many SNPs have been detected in many different crops, and these SNPs are useful to discriminate between closely related individuals, fine mapping of QTLs, characterization of genes contributing to quantitatively inherited traits, and to distinguish between closely related QTLs of different traits [8, 9]. In pea, 20,008 and 248,617 SNPs have been identified by genome wide transcriptome sequencing [10] and DNA sequencing [2] of diversity panels, respectively. These SNPs have been used to develop 1536 [10] and 13,200 [2] SNP arrays, and to generate linkage maps and association mapping analyses in different pea populations. Some of these maps have been combined to build consensus maps to increase mapping resolution and genome coverage [10–12]. Genotyping-by-sequencing (GBS) method allows simultaneous SNP discovery and genotyping of populations [13]. In the last few years, GBS has been used for SNP identification, generation of high-density linkage maps, and fine mapping of QTLs for various traits in a diverse range of crops [14–16], including mapping of Ascochyta blight resistance [17] and components of heat stress resistance [18] in pea. Recently, Boutet et al. [19] identified 419,024 SNPs using HiSeq whole genome sequencing of four pea lines. A subset of 64,263 markers were genotyped in a subpopulation of 48 RILs of a mapping population using GBS and a genetic linkage map was constructed [19]. The currently available sequencing and genotyping technologies can be used to accelerate breeding for key traits by exploiting the diversity of pea germplasm, identification of trait-linked markers, and their use in marker-assisted selection (MAS).

Bi-parental mapping populations are useful for high precision mapping of various traits [20], and the markers thus identified have been used for MAS. Determining marker-phenotype association for complex traits is a difficult task because of the number of underlying QTLs, QTL x Environment interaction, and epistasis [21]. Therefore, multi-location, multi-year replicated field trials are required to characterize QTLs associated with complex traits. QTLs responsible for the genetic control of various biotic stresses, seed protein content, lodging resistance, and seed nutrition in pea have been reported [2, 6, 22]. The current study was designed to identify QTLs for Mycosphaerella blight resistance, lodging resistance, agronomic traits, seed mineral concentration, fibre concentration, and grain yield using three bi-parental populations of pea, repeated phenotyping at two different field locations, and high density genetic linkage mapping by GBS, for MAS in pea breeding.

## Methods

### Plant materials

Three recombinant inbred populations (RILs) of pea, namely, PR-02 derived from the cross Orb/CDC Striker, PR-07 derived from the cross Carrera/CDC Striker, and PR-15 derived from the cross 1–2347-144/CDC Meadow, each consisting of 94 individuals were used for phenotyping and genotyping. Cultivar Orb was developed by Sharpes (UK), cultivar Carrera was developed by Limagrain (The Netherlands), cultivar CDC Striker [23], cultivar CDC Meadow [24], and breeding line 1–2347-144 [25] were developed by the Crop Development Centre, University of Saskatchewan, Canada. All the RIL populations were developed at the Crop Development Centre, University of Saskatchewan using single seed descent to the F5 generation. A single seed of each RIL at F_9_ generation was used for genotyping, and PR-02 and PR-07 RILs of F_6_ to F_8_ generations, and PR-15 RILs of F_7_ and F_8_ generations, were used for phenotyping.

### Genotyping-by-sequencing (GBS)

RIL populations were genotyped using GBS according to the protocol described in detail by Elshire et al. [13].

#### Library preparation and sequencing

Young leaf tissue harvested and freeze dried from ~ 14-day old seedlings was used for DNA extraction using the QIAGEN DNeasy 96 plant kit. DNA was quantified using picogreen and DNA concentration of each RIL was normalized to 20 ng/μl. Two hundred ng of DNA of each individual was digested with restriction enzymes *Pst*I and *Msp*I, and ligated with unique 4–8 sequence barcode adapters. From the 40 μL individual ligation reaction mixture, five μL of adapter ligated DNA of 47 RILs and 15 μL of adapter ligated DNA of each parent were pooled separately in a single tube to produce 49-plex libraries. The pooled DNA was PCR-amplified using sequencing primer followed by purification using a QIAGEN PCR purification kit. The purified DNA library was quantified using a Bioanalyzer (Agilent Technologies) and GBS 49-plex libraries were sequenced on a single lane of Illumina HiSeq™ 2500 platform (Illumina® Inc., San Diego, CA, USA) using V4 sequencing chemistry at SickKids Hospital, Toronto, Canada.

#### SNP variant calling

From the Illumina data of each library, sequences were assigned to individual samples based on the 4 to 8 base pair barcode adapters ligated to individual DNA using in-house Perl scripts. Following deconvolution, barcode sequences were removed from the sequences, and the reads were trimmed for quality using the read trimming tool Trimmomatic-0.33. To discover polymorphisms, filtered reads were mapped to the draft genome assembly provided through the international pea genome sequencing consortium (unpublished) using the sequence alignment tool Bowtie 2 version 2.2.5. Samtools-1.1 and BCFtools-1.1 were utilized to call variants and output them in variant call format (VCF) format.

### Construction of genetic linkage maps

Polymorphic SNP markers from the previously published linkage maps of the three RIL populations (Sindhu et al., 2014) were included in the linkage analysis along with the polymorphic markers identified by GBS in the current study. Polymorphic markers with less than 15% of missing data per sample were used to construct the genetic linkage maps. The segregation distortion of each marker was assessed by Chi-square test and markers with a distortion probability of greater than 90% were omitted from the linkage analysis. The linkage analysis was conducted using MstMap program with a logarithm of odds (LOD) value of 9.0 and Kosambi mapping function (26, 27).

### Phenotyping of mapping populations

PR-02 and PR-07 were phenotyped in two location field trials over three years, and PR-15 was phenotyped in two location field trials over two years, from 2010 to 2013 (Tables S1, S2, and S3). The mapping populations were evaluated for agronomic traits plant height, days to flower, days to maturity and lodging (1–9 rating scale, 1 = no lodging to 9 = completely lodged at physiological maturity), grain yield and 1000 seed weight according to methods reported in Warkentin et al. [3]. PR-02 and PR-07 were evaluated for seed mineral concentration (Fe, Zn and Se) according to methods reported in Diapari et al. [28]. PR-07 was evaluated for acid detergent fiber, neutral detergent fiber, and starch concentration according to methods reported in Arganosa et al. [29]. PR-15 was evaluated for seed phytate concentration according to the methods reported in Warkentin et al. [25]. PR-02 and PR-07 were evaluated for Mycosphaerella blight severity on a 0–9 rating scale (0 = no disease to 9 = completely blighted) from mid-flowering to physiological maturity stages according to Jha et al. [30], and the area and under disease progress curve (AUDPC) was calculated. Seed shape (1 = completely round to 5 = blocky) and seed dimpling (percent of seeds with dimpled seed coat) were evaluated according to the methods reported in Ubayasena et al. [31].

The two locations used for field trials were Sutherland (near the city of Saskatoon) (52°12’ N, 106°63’ W; Dark Brown Chernozem) and Rosthern (52°66’ N, 106°33’ W; Orthic Black Chernozem) in Saskatchewan, Canada. At each location, the RILs were planted in three row plots with 30 cm row spacing, 1 m row length, at a seeding rate of 75 seeds per plot in a randomized complete block design, which was fully replicated two times. Agronomic best management practices as per provincial government and pulse grower manuals for field pea production in Saskatchewan were utilized including appropriate seeding dates, seeding methods, weed control, and harvest methods. The frequency distribution of the trait measurements in each population were tested for normality by the Shapiro-Wilk test [32]. In case the W value was low, the trait was removed from further analysis. ANOVA was conducted for each trait in each location using SAS Mixed Procedure (SAS Institute, 2015). Pearson correlation coefficient values were calculated to determine the correlation of traits measured in each population.

### QTL mapping

The phenotypic means by location for each trait were used for QTL mapping. QTL mapping was performed using composite interval mapping (CIM) using QTL Cartographer v2.5 [33]. To declare a QTL, the threshold for each search was obtained from 1000 permutations with a significance level of 0.05.

## Results

### Genotyping-by-sequencing (GBS)

In all of the sequencing runs of 49-plex libraries approximately 225 million raw reads per lane were obtained from sequencing on Illumina HiSeq™ 2500 platform. Across the three populations, the average read count obtained per RIL was 3.9–4.6 million and 84% of the reads survived after trimming for quality. The trimmed reads were mapped to the reference genome and the number of mapped reads per RIL in PR-02, PR-07 and PR-15 were 3.24, 2.42 and 2.48 million, respectively (Table 1).

**Table 1:** Summary of average read count from sequencing of three mapping populations (49-plex libraries) using genotyping-by-sequencing method

RIL Population	Average number of reads/RIL (millions)			
	Read count	Reads surviving after trimming	No. of unique reads	Total No. of reads mapped to draft genome
PR-2 (Orb x CDC Striker)	4.6	4.02	1.15 (28.7%)	3.24 (80.8%)
PR-7 (Carerra x CDC Striker)	4.0	3.15	1.08 (34.3%)	2.42 (76.8%)
PR-15 (1–2347-144 x CDC Meadow)	3.9	3.19	1.19 (37.3%)	2.48 (77.8%)

### Genetic linkage mapping

Of the > 25,000 SNPs identified by GBS in each RIL population, after filtering for read depth of 5, percentage of missing values as less than 15%, Chi-square value of > 0.1 probability for segregation distortion, 2066, 3023, and 3444 SNPs were used to construct genetic linkage maps of PR-02, PR-07 and PR-15, respectively. A total of 306, 366, and 337 SNP markers earlier genotyped using Illumina GoldenGate array in the same three populations (Sindhu et al. 2014) were added to the GBS data set for linkage map construction.

**Fig. 1:**
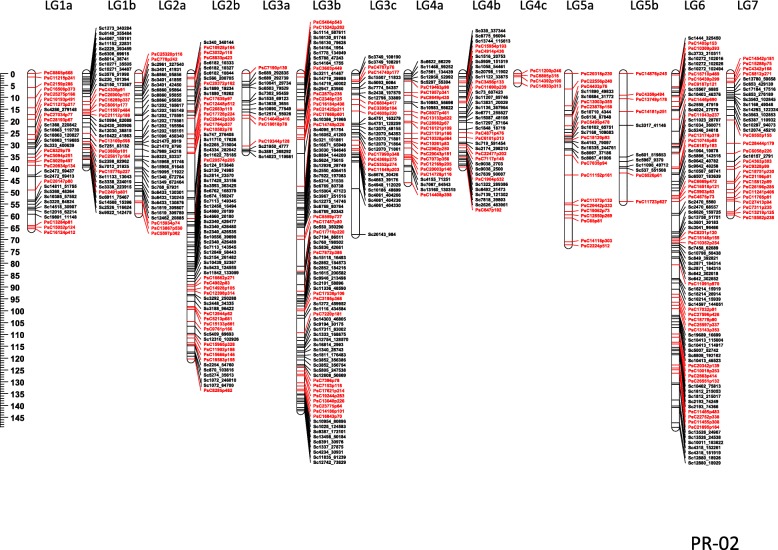
Linkage map of PR-02 (Orb x CDC striker) with 527 non-redundant loci each represented by a group of co-segregating markers. The loci represented by markers in red were reported earlier by Sindhu et al. (2014) and the loci represented by markers in black were added from the current GBS genotyping of the same population

In PR-02, 1866 SNP markers (1560 from GBS and 306 from GoldenGate assay) formed 14 linkage groups with a map length of 951.9 centiMorgans (cM). The length of individual linkage groups ranged from 4.0 to 149.2 cM. The total number of non-redundant loci represented by the mapped SNPs are 527 with 4 to 87 loci per linkage group. The SNP markers identified through GBS represented an additional 329 loci compared to the 198 loci represented by the SNP markers identified using the GoldenGate assay (Sindhu et al. 2014). The average distance between two loci is 1.81 cM and the maximum distance is 19.3 cM (Fig. 1).

**Fig. 2:**
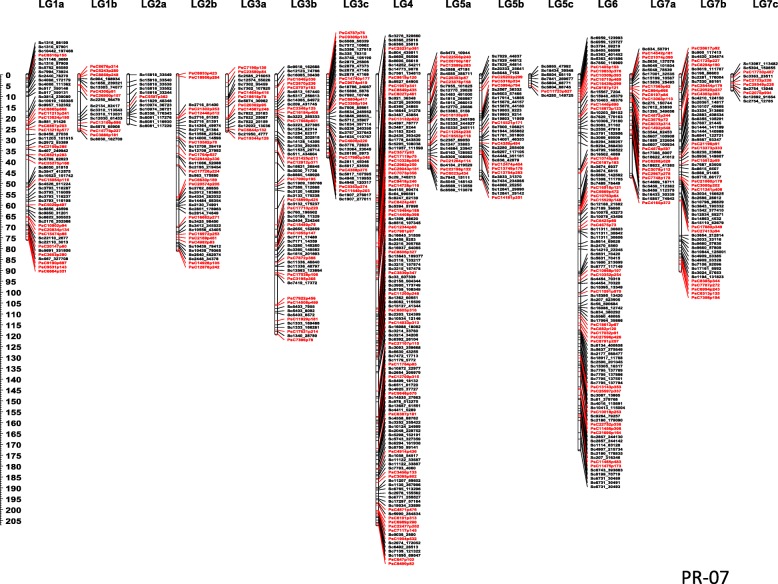
Linkage map of PR-07 (Carrera x CDC striker) representing 675 non-redundant loci each represented by a group of co-segregating markers in 15 linkage groups. The loci represented by markers in black are the result of GBS genotyping compared to the earlier identified loci from SNP genotyping using GoldenGate arrays (Sindhu et al. 2014)

The PR-07 linkage map has 3355 SNP markers (2990 from GBS and 365 from the GoldenGate assay) in 15 linkage groups. These markers together represented a total map distance of 1008.8 cM with a minimum of 7.1 cM and a maximum of 207.0 cM distance per linkage group. A total of 675 non-redundant loci were mapped with a minimum of 10 and maximum of 128 non-redundant loci per linkage group. The SNPs identified through GBS represented 459 non-redundant loci in addition to the 216 loci mapped from previous reported SNPs (Fig. 2).

**Fig. 3:**
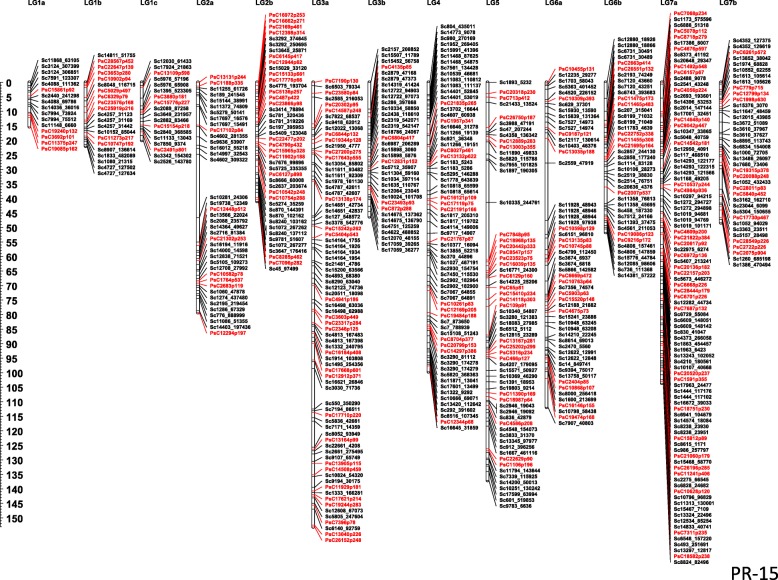
Linkage map of PR-15 (1–2347-144 x CDC Meadow) with 609 non-redundant loci each represented by a group of co-segregating markers. The loci represented by markers in red are the ones identified by Sindhu et al. (2014) and the loci represented by markers in black were identified in the current study through GBS genotyping

The PR-15 linkage map has 3408 SNPs (3077 SNPs from the current GBS and 337 from the GoldenGate assay) grouped into 12 linkage groups. The total map length is 914.2 cM with individual linkage groups ranging from 20.0 cM to 153.1 cM. The map represented 609 non-redundant loci with a minimum of 16 and maximum of 94 per linkage group. Of the total non-redundant loci mapped, 417 are represented by the SNPs identified by GBS and 192 have a representation of earlier genotyped SNPs. The average distance between the non-redundant loci across the linkage groups is 1.5 cM (Fig. 3).

### Phenotypic data

Phenotypic data collected for PR-02, PR-07, and PR-15 are summarized in Additional files 1, 2, and 3, respectively. Data for all traits were tested for normality using the Shapiro-Wilk test. In the vast majority of cases the W value was > 0.90. In cases in which the W value was low, the traits were removed from further analysis.

All three populations were assessed for days to flowering. In the case of PR-02 and PR-15, RILs flowered within a relatively narrow range, while this range was generally greater, approximately one week, for PR-07 depending on the station/year. The range in days to maturity was wider for all three RIL populations, generally from one to two weeks. Substantial variation was noted in all three RIL populations for plant height and lodging score, with greatest variation in PR-07 for both traits. Similarly for grain yield, substantial variation was noted in all three RIL populations, with somewhat greater range in variation noted in PR-07 and PR-15. Mean yield ranged from 195 to 516 g m^− 2^ in PR-02, 266–536 g m^− 2^ in PR-07, and 307–858 g m^− 2^ in PR-15. Mean commercial yield of field pea in western Canada is approximately 270 g m^− 2^, indicating that the three RIL populations have good yield potential, and that the field trials reported here were conducted under conditions favourable for expression of yield potential. The Mycosphaerella blight resistance, reported as AUDPC ranged from 156 to 208 in PR-07 RILs measured at all six station/years.

Several post-harvest seed quality traits were assessed in some of the RIL populations. Substantial variation was noted in 1000 seed weight, as well as protein, iron, zinc, and selenium concentration in PR-02 and PR-07. In the case of seed selenium concentration, the range in variation among RILs in PR-02 and PR-07 was greater at the Sutherland (Saskatoon) site than at the Rosthern site in each year. Peas grown in the Saskatoon region are known to have generally greater selenium concentration than peas grown in the Rosthern region [34], and that was also observed in this research. Acid detergent fiber, neutral detergent fiber, and starch concentrations of PR-07 lines varied significantly at each station/year assessed, as did seed coat dimpling and seed shape. PR-15 was developed from the cross between a low phytate and a normal phytate parent, and as expected, lines differed significantly for this trait at each station/year assessed.

Correlation analyses in the three RIL populations were presented in Additional files 4, 5, and 6. In PR-02 and PR-07, the strongest correlation was between days to flowering and days to maturity, followed by correlation between days to maturity and yield in PR-02, and Mycosphaerella blight severity and lodging in PR-07. In PR-15, the strongest correlation observed was between plant height and yield, followed by plant height and days to maturity, days to maturity and yield, and days to flowering and lodging resistance.

### QTL detection in pea mapping populations

Across the three pea mapping populations, a total of 375 QTLs, i.e., 96 in PR-02, 225 in PR-07, and 54 in PR-15, were identified for multiple traits in multiple environments. The identified QTLs had a maximum LOD value of 23.8 and explained phenotypic variation of up to 62.9% in case of QTLs identified for plant height in PR-07. Of all the identified QTLs, 292 trait specific QTLs were detected on the same linkage group in more than one environment within the same population (Tables 2, 3 and 4).

**Table 2:** Quantitative trait loci (QTL) identified for multiple traits in PR-02 mapping population

Trait	Station/year	Linkage group													
		LG1a	LG1b	LG2a	LG2b	LG3a	LG3b	LG3c	LG4a	LG4b	LG4c	LG5a	LG5b	LG6	LG7
Days to Flowering	2011 Sutherland			4.46										5.29	
	2011 Rosthern											3.54		9.16	
	2012 Sutherland					3.6								12.84	
	2012 Rosthern											3.79			
	2013 Sutherland				5.25							3.86		19.0	
	2013 Rosthern													10.63	
Days to Maturity	2011 Sutherland						4.26							5.56	
														8.19	
	2011 Rosthern						4.46							3.19	
							4.8								
	2012 Sutherland									5.06					
	2012 Rosthern						7.69					3.06	6.33		
	2013 Sutherland													5.0	
	2013 Rosthern			3.07				4.1						8.4	
Plant height (cm)	2011 Sutherland						12.81		6.45						
							8.1								
	2011 Rosthern						5.09							4.56	
	2012 Sutherland			6.2			5.47					4.05			
				4.41											
	2012 Rosthern						16.71					4.22			
	2013 Rosthern	4.43													
Lodging Resistance (1–9)	2011 Sutherland												3.73		
	2011 Rosthern						3.92								
							3.61								
	2012 Sutherland														
	2012 Rosthern						8.83								
	2013 Rosthern											4.64			
Yield (g/m^2^)	2011 Sutherland								22.31					3.09	
														3.26	
	2011 Rosthern						3.38		10.99					6.2	
														3.54	
	2012 Sutherland													4.73	
	2012 Rosthern						3.2		11.24						
	2013 Sutherland													6.61	
	2013 Rosthern							3.48						7.96	
1000 seed weight (g)	2011 Sutherland	3.06	3.4				3.03		6.15					4.64	
	2011 Rosthern						4.6			4.46		5.51		3.61	
Seed protein conc. (%)	2012 Sutherland											3.17			
	2013 Sutherland		5.00						3.39						
	2013 Rosthern		4.97						3.37						
Acid detergent Fiber (%)	2012 Sutherland									4.64		6.86			
	2013 Sutherland													3.44	8.36
	2013 Rosthern														8.65
Neutral detergent Fiber (%)	2012 Sutherland									3.53		5.5			
	2013 Sutherland											5.05			6.07
	2013 Rosthern											5			5.96
Seed starch conc. (%)	2012 Sutherland								4.44						
	2013 Sutherland				5.54										
	2013 Rosthern				5.53										
Seed iron conc. (ppm)	2011 Sutherland				3.97		7.61			3.34					
	2011 Rosthern						6.28							3.37	
Seed selenium conc. (ppm)	2011 Sutherland								11.6			3.51			3.2
	2011 Rosthern														3.1
Seed zinc conc. (ppm)	2011 Sutherland	4.32													
	2011 Rosthern						13.71							4.52	
														4.52	

Values presented in the table represent logarithm of the odds (LOD) of the QTL peak

**Table 3:** Quantitative trait loci (QTL) identified for multiple traits in PR-07 mapping population

Trait	Station/year	Linkage group														
		LG1a	LG1b	LG2a	LG2b	LG3a	LG3b	LG3c	LG4	LG5a	LG5b	LG5c	LG6	LG7a	LG7b	LG7c
Days to flowering	2010 Sutherland	3.41														
		4.13														
	2010 Rosthern	5.52					3.09		3.00				5.10			
	2011 Sutherland				4.72		4.99									
	2011 Rosthern						3.00									
							4.80									
	2012 Sutherland															
	2012 Rosthern						4.13									
Days to Maturity	2010 Sutherland	4.35					4.02		7.90							
	2010 Rosthern							3.77						3.37		
	2011 Sutherland					4.14								3.24		
	2011 Rosthern												4.39			
	2012 Sutherland	3.85									3.41					
	2012 Rosthern						4.29									
							3.54									
Plant height (cm)	2010 Sutherland						15.74		3.75							
	2010 Rosthern				3.06		23.8									
	2011 Sutherland				3.3		11.89									
	2011 Rosthern						16.17									
		3.25														
	2012 Sutherland	4.26					23.82							7.15		
	2012 Rosthern						20.52							5.09		
Mycosphaerella resistance (AUDPC)	2010 Sutherland						3.89		4.78							
	2010 Rosthern										3.21		5.96			
	2011 Sutherland						7.76		4.06		6.31					
	2011 Rosthern															
	2012 Sutherland	3.23	3.15				3.54									
	2012 Rosthern						8.85							6.24		
Lodging resistance (1–9)	2010 Sutherland						13.19	3.92								
	2010 Rosthern						6.85						7.21			
													6.52			
	2011 Sutherland						3.43									
	2011 Rosthern														4.25	
	2012 Sutherland				4.73		4.07						4.07			
	2012 Rosthern						6.43							10.3		
Yield (g/m^2^)	2010 Sutherland						9.24		3.02					5.58		
	2010 Rosthern															
	2011 Sutherland						3.75							3.1		
	2011 Rosthern						14.13									
	2012 Sutherland						6.79		3.1					3.98		
	2012 Rosthern						4.02		4.12							
							4.08									
1000 seed weight (g)	2010 Sutherland							14.51	3.83	4.01			4.35			
	2010 Rosthern						3.41	11.38	4.00				3.75			
									6.84							
	2011 Sutherland						4.97		3.05							
	2011 Rosthern							11.69	3.05				3.33			
	2012 Sutherland							3.07					3.33	6.62		
	2012 Rosthern							15.57		5.58						
Seed protein conc. (%)	2010 Sutherland						3.16						3.15			
	2010 Rosthern														3.88	
	2011 Sutherland												6.21		3.16	
	2011 Rosthern	3.22		3.42	6.51		3.84	6.01						4.56		
	2012 Sutherland	3.98												4.83	3.14	
	2012 Rosthern						4.08				3.11					
Acid detergent fibre (%)	2010 Sutherland												3.84			
	2010 Rosthern	15.74							6.42							
									6.55							
	2011 Sutherland						10.1		4.74					4.95		
	2011 Rosthern						3.87		4.92					5.15		
	2012 Sutherland	12.07			3.2								3.39	5.32		
	2012 Rosthern				4.05				6.22				3.18			3.41
Neutral detergent fibre (%)	2010 Sutherland	3.72			8.61											
	2010 Rosthern	4.76														
	2011 Sutherland	7.25					7.44		5.97					5.05		
		3.32														
	2011 Rosthern	13.61							5.84					3.63		
	2012 Sutherland	19.46							6.95		3.05			5.12		
	2012 Rosthern	14.72							6.94							
Seed starch conc. (%)	2010 Sutherland															
	2010 Rosthern						4.03							3.33		
	2011 Sutherland	6.09						4.39								
		5.03														
	2011 Rosthern	6					7.05	3.4						8.39		
	2012 Sutherland	7.57					3.27	4.73						6.6		
	2012 Rosthern	5.79														
	2012 Rosthern	7.56					6.37	3.76						3.56		
Seed iron conc. (ppm)	2010 Sutherland						4.13							4.22		
	2010 Rosthern						10.14		3.62							
							5.84									
	2011 Sutherland						2.89		7.14							
	2011 Rosthern						4.56					3.03				
	2012 Sutherland						4.04				3.28					
	2012 Rosthern					3.18	10.05							6.93		
Seed selenium conc. (ppm)	2010 Sutherland				3.65					3.68						
	2010 Rosthern										3.08					
											4.51					
	2011 Sutherland								3.12					3.29		
									3.81							
	2011 Rosthern														4.32	
	2012 Sutherland										4.15					
	2012 Rosthern															
Seed zinc conc. (ppm)	2010 Sutherland						4.48							5.85		
							17.3									
	2010 Rosthern						17.11							3.67		
	2011 Sutherland		4.44				6.61									
	2011 Rosthern						4.09									
							12.09									
	2012 Sutherland				4.61				4.9							
	2012 Rosthern	3.46					4.48									
							8.56							4.61		
Seed dimpling (%)	2010 Sutherland						3.69			8.85						
	2010 Rosthern							3.62		9.14						
	2011 Sutherland									9.02						
	2011 Rosthern									4.12						
	2012 Sutherland									9.53						
	2012 Rosthern							5.99	4.85	10.37						
Seed shape (1–5)	2010 Sutherland				3.96			8.3						7.87		
	2010 Rosthern				6.02			8.57	5.52							
	2011 Sutherland							4.56					3.68			
	2011 Rosthern							8.29					4.24	6.48		
	2012 Sutherland						4.72	3.71						6.29		
	2012 Rosthern							7.65						8.46		

Values presented in the table represent logarithm of the odds (LOD) of the QTL peak

**Table 4:** Quantitative trait loci (QTL) identified for multiple traits in PR-15 mapping population

Trait	Station/year	Linkage group												
		LG1a	LG1b	LG1c	LG2a	LG2b	LG3a	LG3b	LG4	LG5	LG6a	LG6b	LG7a	LG7b
Days to flowering	2012 Sutherland								3.8					
	2012 Rosthern						4.69			5.21	3.52			
	2013 Sutherland					4.48	4.58			7.22				
	2013 Rosthern									3.94			3.73	
Days to maturity	2012 Sutherland		3.99				3.51			3.57				
	2012 Rosthern						4.71			3.2				
							3.33							
	2013 Sutherland					4.02	3.76			6.55			5.01	
	2013 Rosthern						5.79			3.64			5.6	
Plant height (cm)	2012 Sutherland						4.35							
	2012 Rosthern						12.99		5.25				5.92	
	2013 Sutherland						6.06			3.22				
							3.47							
	2013 Rosthern				5.2		12.94							
Lodging resistance (1–9)	2012 Sutherland						9.15							
	2012 Rosthern						8.83						3.11	
	2013 Sutherland										3.37			
	2013 Rosthern					3.7								
Yield (g/m^2^)	2012 Sutherland		3.39				5.45							
	2012 Rosthern		3.16				4.1		9.06					
	2013 Sutherland						4.67							
							3.11							
	2013 Rosthern						4.77							
Seed phytate conc. (ppm)	2012 Sutherland						3.52			10.59	5.09			
											6.93			
	2012 Rosthern									12.48				
	2013 Sutherland						4.08			11.37	4.53			
											6.03			
	2013 Rosthern									6.69				

Values presented in the table represent logarithm of the odds (LOD) of the QTL peak

#### QTLs for agronomic traits

##### QTLs for days to flowering

Of the multiple QTLs identified for DTF, QTLs on LG5a and LG6 in PR-02, LG1a and LG3b in PR-07, LG3a and LG5 in PR-15 were identified in multiple trials (Tables 2, 3 and 4). Compared between the three populations and environments tested, the QTL on LG6 in PR-02 was significant in five of the six trials. In all the five trials, the QTL position shared a common position on the same linkage group. The LOD values of this QTL on LG6 ranged from 5.3–19.0 and explained a phenotypic variance of 17.9 to 47.9% (Table 5).

**Table 5:** Details of quantitative trait loci identified for different traits in PR-02 population in repeated tests

Linkage group	Trait	Station/year	QTL interval (cM)	Flanking markers	LOD	Add. effect^a^	% phenotypic variance explained
LG1a	Seed zinc conc. (ppm)	2011 Sutherland	22.0–31.7	Sc4286_279148 - Sc10863_119685	4.32	−0.77	12.72
LG1b	Seed protein conc. (%)	2013 Sutherland	28.0–29.9	Sc7251_83132 - Sc2398_82962	5.00	−0.43	14.55
LG1b	Seed protein conc. (%)	2013 Rosthern	28.1–29.9	Sc7251_83132 - Sc2398_82962	4.97	−0.43	15.85
LG2b	Seed starch conc. (%)	2013 Sutherland	1.8–24.9	Psc16928p164 - Psc12448p512	5.54	0.49	21.03
LG2b	Seed iron conc. (ppm)	2011 Sutherland	3.9–6.4	PsC3032p118 - Sc6182_10333	3.97	−1.00	12.55
LG2b	Seed starch conc. (%)	2013 Rosthern	6.6–10.6	Sc6182_10333 - Sc6182_10844	5.53	0.49	21.00
LG3b	1000 seed weight (g)	2011 Sutherland	1.1–4.1	Psc5404p543 - Sc16130_61478	3.03	−4.96	6.31
LG3b	Days to maturity	2011 Rosthern	2.0–5.9	PsC15242p262 - Sc14164_1954	4.46	1.73	6.97
LG3b	Days to maturity	2011 Sutherland	14.9–22.2	PsC3603p449 - PsC2870p236	4.26	−0.378	7.75
LG3b	Days to maturity	2011 Rosthern	15.8–18.1	Sc3211_41467 - Sc14719_40002	4.80	−1.35	22.87
LG3b	Plant height (cm)	2011 Sutherland	33.8–50.1	Sc10386_31966 - Sc4095_91754	12.81	−3.99	30.09
LG3b	Seed iron conc. (ppm)	2011 Rosthern	47.4–58.7	PsC14749p226 - Sc5214_31535	6.28	−1.03	20.72
LG3b	Seed iron conc. (ppm)	2011 Sutherland	47.5–58.6	PsC14749p226 - Sc5214_31535	7.61	−1.47	26.67
LG3b	1000 seed weight (g)	2011 Rosthern	49.7–54.3	PsC14749p226 - Sc5624_75165	4.57	−6.84	11.62
LG3b	Plant height (cm)	2011 Rosthern	69.9–83.1	PsC17487p80 - PsC17710p220	5.09	−2.37	17.7
LG3b	Days to maturity	2012 Rosthern	70.0–91.3	PsC17487p80 - PsC7872p386	7.69	−0.49	24.92
LG3b	Plant height (cm)	2012 Rosthern	70.0–91.3	PsC17487p80 - PsC7872p386	16.71	−1.88	41.54
LG3b	Plant height (cm)	2012 Sutherland	73.2–91.6	PsC17487p80 - PsC7872p386	5.47	−1.10	18.17
LG3b	Plant height (cm)	2011 Sutherland	88.5–106.2	Sc5836_42661 - PsC7220p181	8.10	−2.89	15.93
LG3b	Seed zinc conc. (ppm)	2011 Rosthern	91.8–109.1	PsC7872p386 - PsC7220p181	13.71	1.22	25.83
LG4a	Seed selenium conc. (ppm)	2011 Sutherland	20.1–35.2	PsC28982p67 - PsC20003p140	11.6	−0.13	18.76
LG4a	Yield (g/m^2^)	2012 Rosthern	20.7–38.4	PsC16121p109 - Sc4155_71251	11.24	−38.24	31.23
LG4a	Yield (g/m^2^)	2011 Sutherland	20.9–31.3	PsC16121p109 - PsC29043p181	22.31	−74.6	54.28
LG4a	Seed protein conc. (%)	2013 Sutherland	24.8–25.8	PsC21191p166 - PsC10328p564	3.39	−0.33	10.23
LG4a	Seed protein conc. (%)	2013 Rosthern	24.8–26.1	PsC16121p109 - PsC10328p564	3.37	−0.33	10.26
LG4b	Seed iron conc. (ppm)	2011 Sutherland	2.3–3.4	Sc13744_115613 - PsC4914p436	3.34	0.94	11.14
LG5a	Neutral detergent fibre (%)	2012 Sutherland	0–6.4	PsC20318p230 - PsC22556p240	5.50	−0.17	19.04
LG5a	Neutral detergent fibre (%)	2013 Sutherland	6.4–19.1	PsC22556p240 - PsC23878p158	5.05	−0.28	16.22
LG5a	Neutral detergent fibre (%)	2013 Rosthern	15.4–17.9	Sc11890_49833 - PsC13003p355	5.00	−0.28	16.41
LG5a	Plant height (cm)	2012 Rosthern	18.3–25.9	PsC13003p355 - Psc5495p478	4.22	−0.8	8.02
LG5a	Seed selenium conc. (ppm)	2011 Sutherland	19.8–31.4	PsC13003p355 - Sc8067_37186	3.51	0.07	8.39
LG5a	Plant height (cm)	2012 Sutherland	20.7–26.0	PsC13003p355 - PsC5495p478	4.05	−0.91	12.32
LG5a	Days to flowering	2012 Rosthern	25.1–26.6	Sc6136_87848 - Sc10102_65751	3.79	0.23	12.39
LG5a	Days to flowering	2011 Rosthern	59.7–66.9	PsC12850p269 - Psc14118p303	3.54	0.20	10.38
LG5a	Days to flowering	2013 Sutherland	60.2–68.2	PsC12850p269 - Psc14118p303	3.86	0.35	7.30
LG6	Seed iron conc. (ppm)	2011 Rosthern	0.2–3.0	Sc1444_325450 - PsC10309p393	3.37	0.75	10.33
LG6	Yield (g/m^2^)	2011 Sutherland	0.5–6.5	PsC1405p153 - Sc10272_102016	3.09	21.41	4.45
LG6	Seed zinc conc. (ppm)	2011 Rosthern	1.6–6.8	PsC1405p153 - Sc10272_102016	4.52	0.61	7.10
LG6	Seed zinc conc. (ppm)	2011 Rosthern	9.3–10.8	Sc10272_102494 - PsC16439p299	4.52	0.61	8.04
LG6	Yield (g/m^2^)	2012 Sutherland	19.3–24.4	PsC1446p690 - Sc2559_47919	3.54	−17.5	14.04
LG6	Yield (g/m^2^)	2012 Sutherland	31.9–38.4	Sc1034_360071 - PsC10745p88	4.73	−22.1	16.18
LG6	Days to flowering	2013 Sutherland	75.3–85.6	Sc2871_184315 - Sc16214_15919	19.0	−0.91	30.9
LG6	Days to flowering	2011 Rosthern	75.3–85.6	Sc2871_184315 - Sc16214_15919	9.16	−0.35	17.88
LG6	Days to flowering	2013 Rosthern	75.3–85.6	Sc2871_184315 - Sc16214_15919	10.63	−0.91	26.19
LG6	Days to flowering	2012 Sutherland	79.9–85.6	PsC11091p970 - Sc16214_15919	12.84	−0.96	47.9
LG6	Yield (g/m^2^)	2011 Rosthern	80.6–90.8	PsC11091p970 - Sc14597_144051	6.20	−26.7	11.82
LG6	Yield (g/m^2^)	2013 Rosthern	80.8–92.9	PsC11091p970 - Psc17032p91	7.96	−33.01	24.62
LG6	Yield (g/m^2^)	2013 Sutherland	81.2–92.8	PsC11091p970 - Psc17032p91	6.61	−33.01	19.38
LG6	Days to maturity	2013 Sutherland	82.1–93.0	PsC11091p970 _Psc17032p91	5.00	−0.69	16.63
LG6	Days to maturity	2011 Sutherland	82.6–92.9	PsC11091p970 - Psc17032p91	5.56	−0.49	10.6
LG6	Days to maturity	2013 Rosthern	82.6–92.9	PsC11091p970 - PsC17032p91	8.4	−0.69	33.38
LG6	Days to flowering	2011 Sutherland	87.9–97.3	Sc16214_15939 - PsC25597p337	5.29	−0.20	17.63
LG6	Days to maturity	2011 Rosthern	96.2–98.5	PsC18778p90 - Psc13143p353	3.19	−0.71	9.17
LG6	1000 seed weight (g)	2011 Rosthern	96.5–98.3	PsC18778p90 - Psc13143p353	3.58	−6.1	8.05
LG6	Days to maturity	2011 Sutherland	123.0–126.3	Psc11465p483 - PsC21695p164	8.19	−0.51	16.37
LG6	Yield (g/m^2^)	2011 Sutherland	123.4–130.5	PsC11465p483 - Sc13526_24967	3.26	−22.19	4.69
LG6	1000 seed weight (g)	2011 Sutherland	1.4–3.4	PsC21695p164 - Sc10011_183622	4.64	−6.89	11.03
LG7	Seed selenium conc. (ppm)	2011 Sutherland	11.0–12.5	Sc14293_122324 - Sc6387_110932	3.20	−0.06	7.14
LG7	Seed selenium conc. (ppm)	2011 Rosthern	14.7–19.5	Sc6387_110932 - PsC6555p155	3.10	−0.04	15.04
LG7	Neutral detergent fibre (%)	2013 Sutherland	40.43 - 52.31	PsC9328p99 - PsC11241p406	6.07	0.31	14.64
LG7	Acid detergent fibre (%)	2013 Sutherland	40.43 - 52.31	PsC9328p99 - PsC11241p406	8.36	0.23	28.08
LG7	Neutral detergent fibre (%)	2013 Rosthern	40.43 - 52.31	PsC9328p99 - PsC11241p406	3.90	0.24	10.03
LG7	Acid detergent fibre (%)	2013 Rosthern	40.43 - 52.31	PsC9328p99 - PsC11241p406	8.65	0.22	26.21

Details of QTLs that were identified on the same linkage group in at least 50% of the trials were only listed

^a^Additive effect. Any positive value of additive effect indicates that the trait alleles are from Orb and the negative additive effect indicates the trait alleles are from CDC Striker

##### QTLs for days to maturity

QTLs for DTM positioned on LG3b and LG6 of PR-02 (Table 2), LG1a and LG3b of PR-07 (Table 3), and LG3a and LG5 of PR-15 (Table 4) were identified in repeated trials. QTLs on LG6 in PR-02 and LG3a in PR-15 shared a common position within these two respective linkage groups. In PR-02, the QTLs on LG6 represented a maximum LOD value of 8.4 and explained 33.4% of phenotypic variance. In the same population, the QTLs on LG3b represented a maximum LOD value of 7.7 and explained a phenotypic variance of up to 24.9% (Table 5). The maximum LOD value of the QTLs identified in PR-07 and PR-15 was 4.4 and 6.6 and these QTLs explained a phenotypic variance of 11.8 and 15.2%, respectively (Tables 3 and 4). These QTLs located on LG1a in PR-07 and LG5 of PR-15 (Table 7) shared the same linkage group positions with the most significant QTLs identified for DTF.

##### QTLs for plant height

Multiple QTLs were identified for plant height in all three mapping populations. However, the QTLs of LG3b in PR-02 and PR-07, and LG3a in PR-15 were identified in all of the trials (Tables 2, 3 and 4). These QTLs also shared linkage group positions between most of the trials in each population, and represented a maximum LOD value of 16.7, 23.8 and 13.0, and explained a phenotypic variance of 41.5, 62.9 and 27.4, in PR-02, PR-07 and PR-15, respectively (Tables 5, 6 and 7).

**Table 6:** Details of quantitative trait loci identified for different traits in PR-07 population in repeated tests

Linkage group	Trait	Station year	QTL interval (cM)	Flanking markers	LOD	Add. effect^a^	% phenotypic variance explained
LG1a	Neutral detergent fibre (%)	2011 Sutherland	0–2.2	Sc1316_98199 - PsC8516p155	7.25	− 0.34	14.03
LG1a	Seed starch conc. (%)	2011 Sutherland	1.5–4.6	Sc1316_97901 - Sc5782_95080	6.09	0.55	16.80
LG1a	Neutral detergent fibre (%)	2010 Rosthern	4.5–5.7	Sc1316_97909 - Sc2440_78270	4.76	0.25	14.72
LG1a	Neutral detergent fibre (%)	2012 Sutherland	5.6–9.5	Sc7994_73924 - Sc517_350146	19.46	−0.67	43.99
LG1a	Seed starch conc. (%)	2011 Rosthern	5.7–9.4	Sc7994_73924 - Sc517_350146	6.00	0.58	14.83
LG1a	Neutral detergent fibre (%)	2012 Rosthern	5.7–9.5	Sc2440_78270 - Sc517_350146	14.72	−0.57	38.76
LG1a	Seed starch conc. (%)	2012 Sutherland	5.7–9.5	Sc7994_73924 - Sc517_350146	7.57	0.58	19.03
LG1a	Neutral detergent fibre (%)	2011 Rosthern	5.8–9.5	Sc7994_73924 - Sc517_350146	13.61	−0.55	32.03
LG1a	Seed starch conc. (%)	2012 Rosthern	5.8–9.5	Sc7994_73924 - Sc517_350146	7.56	0.70	17.77
LG1a	Neutral detergent fibre (%)	2010 Sutherland	36.7–38.4	PsC8521p283 - PsC25275p166	3.72	0.54	11.01
LG1a	Neutral detergent fibre (%)	2011 Sutherland	68.9–69.7	Sc22110_2677 - PsC20147p80	3.32	−0.23	6.36
LG1a	Seed starch conc. (%)	2011 Sutherland	71.5–74.4	Sc5691_331806 - PsC9531p143	5.03	−0.52	13.51
LG3b	Days to flowering	2012 Rosthern	0–2.0	Sc9618_162688 - Sc12123_74766	4.13	−0.43	14.54
LG3b	Mycosphaerella resistance^b^	2010 Sutherland	9.2–12.6	PsC1846p336 - Sc5317_256613	3.89	3.75	10.46
LG3b	Lodging resistance (1–9)	2012 Sutherland	32.1–39.7	PsC21425p211 - Sc3030_71736	4.07	0.28	13.59
LG3b	Plant height (cm)	2011 Rosthern	40.2–50.7	Sc3030_71736 - PsC7000p195	16.17	−0.83	46.36
LG3b	Plant height (cm)	2012 Rosthern	40.2–50.7	Sc3030_71736 - PsC7000p195	20.52	−9.90	50.43
LG3b	Mycosphaerella resistance	2012 Rosthern	40.4–50.6	Sc3030_71736 - PsC7000p195	8.85	10.60	26.29
LG3b	Mycosphaerella resistance	2011 Sutherland	41.0–50.6	Sc3030_71736 - PsC7000p195	7.76	5.60	22.12
LG3b	Days to flowering	2011 Sutherland	44.5–50.6	Sc3030_71736 - PsC7000p195	4.99	−0.30	12.49
LG3b	Mycosphaerella resistance	2012 Sutherland	49.5–52.5	Sc8865_149928 - Sc7388_112888	3.54	−4.53	10.70
LG3b	Seed iron conc. (ppm)	2012 Rosthern	53.1–68.7	Sc3120_148299 - PsC18899p425	10.05	−2.64	35.52
LG3b	Seed iron conc. (ppm)	2011 Rosthern	53.2–68.8	Sc3120_148299 - PsC18899p425	4.56	−1.59	17.69
LG3b	Seed iron conc. (ppm)	2011 Sutherland	55.8–68.6	Sc3120_148299 - PsC18899p425	2.89	−1.10	10.85
LG3b	Plant height (cm)	2012 Sutherland	61.3–71.0	Sc3132_175238 - Sc3132_175237	23.82	−14.00	56.74
LG3b	Plant height (cm)	2011 Sutherland	61.3–71.1	Sc3132_175238 - Sc3132_175237	11.89	−7.87	25.63
LG3b	Yield (g/m^2^)	2010 Sutherland	61.5–71.1	Sc3132_175238 - Sc3132_175237	9.24	88.50	27.38
LG3b	Seed starch conc. (%)	2011 Rosthern	61.5–71.2	Sc3132_175238 - Sc3132_175237	7.05	0.66	17.22
LG3b	Yield (g/m^2^)	2012 Sutherland	61.8–71.1	Sc3132_175238 - Sc3132_175237	6.79	40.50	22.62
LG3b	Lodging resistance (1–9)	2010 Rosthern	62.8–71.1	Sc3132_175238 - Sc3132_175237	6.85	0.55	19.34
LG3b	Seed iron conc. (ppm)	2010 Sutherland	63.0–70.6	Sc3132_175238 - Sc3132_175237	4.13	−1.94	15.43
LG3b	Seed zinc conc. (ppm)	2012 Rosthern	63.8–70.8	Sc3132_175238 - Sc3132_175237	4.48	−1.42	12.98
LG3b	Seed Protein conc. (%)	2011 Rosthern	63.9–70.6	Sc3132_175238 - Sc3132_175237	3.84	−0.33	10.09
LG3b	Seed zinc conc. (ppm)	2010 Sutherland	64.4–70.8	Sc3132_175238 - Sc3132_175237	4.48	−1.48	9.06
LG3b	Seed starch conc. (%)	2012 Sutherland	67.1–71.1	PsC18899p425 - Sc3132_175237	3.27	0.43	7.94
LG3b	Seed iron conc. (ppm)	2010 Rosthern	71.0–77.2	Sc3132_175237 - Sc2404_224246	10.14	−3.26	34.14
LG3b	Plant height (cm)	2010 Rosthern	71.1–73.9	Sc3132_175237 - Sc760_198502	23.80	−9.00	62.90
LG3b	Seed starch conc. (%)	2010 Rosthern	71.1–74.4	Sc3132_175237 - Sc760_198502	4.03	0.40	9.62
LG3b	Plant height (cm)	2010 Sutherland	71.1–74.8	Sc3132_175237 - Sc10190_11329	15.74	−5.67	42.98
LG3b	Seed zinc conc. (ppm)	2011 Rosthern	71.2–74.6	Sc3132_175237 - Sc10190_11329	4.09	−1.11	9.93
LG3b	Lodging resistance (1–9)	2010 Sutherland	71.2–74.8	Sc3132_175237 - Sc10190_11329	13.19	0.59	35.29
LG3b	Yield (g/m^2^)	2011 Sutherland	71.7–74.4	Sc3132_175237 - Sc760_198502	3.75	32.21	10.88
LG3b	Days to flowering	2011 Rosthern	71.9–73.8	Sc3132_175237 - PsC17710p220	3.00	0.82	8.62
LG3b	Lodging resistance (1–9)	2011 Sutherland	72.9–74.7	Sc3132_175237 - Sc10190_11329	3.43	−0.52	11.19
LG3b	Yield (g/m^2^)	2011 Rosthern	77.5–80.3	Sc2404_224246 - Sc7171_17480	14.13	91.50	41.71
LG3b	Seed starch conc. (%)	2012 Rosthern	77.7–80.7	Sc2404_224246 - Sc7171_14359	6.37	0.68	14.54
LG3b	Seed Protein conc. (%)	2012 Rosthern	78.8–79.6	Sc2655_152859 - Sc7171_17480	4.08	−0.56	15.26
LG3b	Yield (g/m^2^)	2012 Rosthern	78.9–79.6	Sc2655_152859 - Sc7171_17480	4.02	46.84	14.62
LG3b	Days to flowering	2010 Rosthern	79.4–80.1	PsC1055p147 - Sc7171_17480	3.09	− 0.79	8.78
LG3b	Yield (g/m^2^)	2012 Rosthern	80.4–81.3	Sc7171_17480 - Sc3260_148260	4.08	34.50	12.22
LG3b	Seed zinc conc. (ppm)	2011 Sutherland	82.3–85.8	Sc7171_14359 - Sc1615_201693	6.61	1.26	19.83
LG3b	Lodging resistance (1–9)	2012 Rosthern	84.8–87.8	Sc3260_148582 - Sc11336_48797	6.43	0.24	16.46
LG3b	Seed zinc conc. (ppm)	2010 Sutherland	87.7–91.0	Sc11336_48840 - PsC17538p106	17.30	3.38	50.13
LG3b	Seed zinc conc. (ppm)	2010 Rosthern	87.9–91.1	Sc11336_48840 - PsC17538p106	17.11	2.60	43.21
LG3b	Seed zinc conc. (ppm)	2011 Rosthern	87.9–91.1	Sc11336_48840 - PsC3195p368	12.09	2.08	34.90
LG3b	Seed zinc conc. (ppm)	2012 Rosthern	88.0–91.0	Sc11336_48840 - PsC17538p106	8.56	2.03	23.92
LG3b	Seed iron conc. (ppm)	2010 Rosthern	89.8–91.8	Sc12583_123864 - Sc7419_17372	5.84	2.29	16.86
LG3b	Days to flowering	2011 Rosthern	89.9–103.8	Sc12583_123864 - PsC7922p456	4.80	−0.58	16.21
LG3b	Seed iron conc. (ppm)	2012 Sutherland	90.7–96.8	Sc12583_123864 - PsC7922p456	4.04	−2.33	17.35
LG3b	Seed Protein conc. (%)	2010 Sutherland	117.2–117.5	PsC17621p214^c^	3.16	0.33	9.61
LG3C	Seed starch conc. (%)	2011 Rosthern	7.1–9.5	Sc3749_108190 - Sc2879_47145	3.40	0.40	7.06
LG3C	Seed starch conc. (%)	2011 Sutherland	7.1–9.5	Sc3749_108190 - Sc2879_47145	4.39	0.46	11.58
LG3C	Seed starch conc. (%)	2012 Sutherland	12.4–15.6	PsC14740p177 - Sc7592_10748	4.73	0.45	11.03
LG3c	Seed shape (1–5)	2011 Rosthern	13.8–16.3	PsC14740p177 - PsC6804p417	8.29	−0.22	22.14
LG3C	1000 seed weight (g)	2010 Sutherland	15.5–18.1	Sc15898_5876 - Sc5634_248952	14.51	−14.20	37.83
LG3C	1000 seed weight (g)	2012 Rosthern	15.5–18.1	Sc15898_5876 - Sc5634_248952	15.57	−18.12	40.02
LG3C	1000 seed weight (g)	2011 Rosthern	15.6–18.9	Sc15898_5876 - Sc3846_98653	11.69	−12.47	32.08
LG3c	Seed shape (1–5)	2012 Rosthern	15.7–17.9	Sc15898_5876 - Sc5634_248952	7.65	− 0.18	17.91
LG3c	Seed shape (1–5)	2011 Sutherland	15.9–17.8	Sc7592_10748 - Sc5634_248952	4.56	−0.15	14.03
LG3C	Seed starch conc. (%)	2012 Rosthern	16.9–17.3	PsC6804p417 - Sc7506_85661	3.76	0.45	8.00
LG3C	1000 seed weight (g)	2012 Sutherland	17.0–17.3	PsC6804p417 - Sc7506_85661	3.07	−5.17	5.73
LG3c	Seed shape (1–5)	2010 Rosthern	18.0–21.4	Sc7506_85661 - Sc1034_397114	8.57	−0.20	19.73
LG3c	Seed shape (1–5)	2012 Sutherland	18.3–21.0	Sc5634_248952 - Sc1034_397114	3.71	−0.13	8.82
LG3c	Seed shape (1–5)	2010 Sutherland	22.5–25.2	Sc5236_240350 - Sc3776_72623	8.30	−0.24	21.88
LG3C	1000 seed weight (g)	2010 Rosthern	29.1–32.8	PsC17990p348 - Sc5017_107595	11.38	−10.80	26.22
LG4	Acid detergent fibre (%)	2010 Rosthern	46.9–54.9	Sc14252_54211 - PsC9619p120	6.42	12.41	12.41
LG4	Acid detergent fibre (%)	2011 Sutherland	47.1–55.1	Sc14773_9078 - PsC9619p120	4.74	0.20	11.43
LG4	Yield (g/m^2^)	2012 Sutherland	55.4–57.3	PsC9619p120 - PsC8649p435	3.10	−24.70	9.68
LG4	Acid detergent fibre (%)	2011 Rosthern	55.4–57.7	PsC9619p120 - PsC8027p461	4.92	0.30	13.80
LG4	1000 seed weight (g)	2010 Rosthern	74.2–78.7	Sc11957_111590 - PsC17119p75	4.00	5.85	7.80
LG4	1000 seed weight (g)	2011 Sutherland	86.2–87.2	PsC2962p250 - Sc29_745213	3.05	7.08	6.80
LG4	Acid detergent fibre (%)	2010 Rosthern	112.4–115.6	Sc16937_64085 - Sc13643_109377	6.55	12.39	12.39
LG4	Neutral detergent fibre (%)	2011 Sutherland	114.0–117.7	PsC8606p327 - Sc3118_133217	5.97	−0.33	12.34
LG4	Neutral detergent fibre (%)	2011 Rosthern	121.0–124.6	PsC3839p347 - Sc6758_108549	5.84	−0.34	12.41
LG4	Acid detergent fibre (%)	2012 Rosthern	125.0–127.9	Sc6758_108549 - Sc15127_41344	6.22	−0.19	18.65
LG4	Neutral detergent fibre (%)	2012 Sutherland	125.0–127.9	PsC11200p246 - Sc15127_41344	6.95	−0.33	11.29
LG4	Neutral detergent fibre (%)	2012 Rosthern	125.1–128.1	PsC11200p246 - PsC6805p316	6.94	−0.36	14.47
LG4	Yield (g/m^2^)	2012 Rosthern	125.3–127.9	PsC11200p246 - Sc15127_41344	4.12	42.50	12.14
LG4	Yield (g/m^2^)	2010 Sutherland	164.1–164.4	PsC5648p575 - Sc14535_27663	3.02	35.90	7.72
LG4	1000 seed weight (g)	2010 Rosthern	171.5–174.8	PsC6387p181 - Sc10124_24989	6.84	7.89	13.98
LG4	1000 seed weight (g)	2010 Sutherland	190.1–192.2	Sc11207_89502 - Sc2978_155562	3.83	6.32	7.43
LG4	1000 seed weight (g)	2011 Rosthern	190.8–192.2	Sc1136_367986 - Sc2978_155562	3.05	5.91	7.26
LG5a	Seed dimpling (%)	2010 Rosthern	2.2–6.2	Sc8473_10944 - PsC12889p283	9.14	5.26	29.19
LG5a	Seed dimpling (%)	2010 Sutherland	2.2–6.2	Sc8473_10944 - PsC12889p283	8.85	6.94	27.10
LG5a	Seed dimpling (%)	2011 Sutherland	2.2–6.2	Sc8473_10944 - PsC12889p283	9.02	3.19	28.03
LG5a	Seed dimpling (%)	2012 Sutherland	2.3–6.3	Sc8473_10944 - PsC12889p283	9.53	5.05	28.71
LG5a	Seed dimpling (%)	2012 Rosthern	2.5–6.3	Sc8473_10944 - PsC12889p283	10.37	8.20	25.69
LG5a	Seed dimpling (%)	2011 Rosthern	2.6–6.2	PsC22556p240 - PsC12889p283	4.12	3.10	12.67
LG6	1000 seed weight (g)	2011 Rosthern	11.4–12.9	PsC16439p299 - Sc15567_7204	3.33	6.07	7.65
LG6	1000 seed weight (g)	2010 Rosthern	118.5–120.3	Sc5637_275649 - Sc2500_201045	3.75	−5.54	7.07
LG6	1000 seed weight (g)	2010 Sutherland	131.5–134.8	PsC25597p337 - Sc10413_115004	4.35	−6.84	8.76
LG6	1000 seed weight (g)	2012 Sutherland	132.0–134.0	Sc3067_13865 - Sc10413_115004	3.33	−4.31	6.43
LG7a	Seed shape (1–5)	2012 Sutherland	0–0.9	Sc634_50791 - PsC21074p390	6.29	0.17	15.70
LG7a	Seed zinc conc. (ppm)	2012 Rosthern	0–1.7	Sc634_50791 - Sc5630_137578	4.61	1.64	11.65
LG7a	Seed zinc conc. (ppm)	2010 Sutherland	0–1.8	Sc634_50791 - Sc9049_101804	5.85	1.61	11.41
LG7a	Neutral detergent fibre (%)	2011 Rosthern	0–1.8	Sc634_50791 - Sc9049_101804	3.63	0.24	6.40
LG7a	Yield (g/m^2^)	2010 Sutherland	0–1.9	Sc634_50791 - Sc9049_101804	5.58	−49.80	15.11
LG7a	Seed shape (1–5)	2011 Rosthern	0–1.9	Sc634_50791 - Sc9049_101804	6.48	−0.19	16.42
LG7a	Neutral detergent fibre (%)	2011 Sutherland	0–1.9	Sc634_50791 - Sc9049_101804	5.05	0.29	9.91
LG7a	Seed shape (1–5)	2010 Sutherland	0–2.0	Sc634_50791 - Sc9049_101804	7.87	0.22	20.62
LG7a	Seed starch conc. (%)	2011 Rosthern	0–2.0	Sc634_50791 - Sc9049_101804	8.39	−0.68	20.10
LG7a	Acid detergent fibre (%)	2011 Sutherland	0–2.0	Sc634_50791 - Sc9049_101804	4.95	0.19	11.27
LG7a	Seed shape (1–5)	2012 Rosthern	0.3–2.6	Sc634_50791 - Sc5048_78539	8.46	0.19	20.23
LG7a	Acid detergent fibre (%)	2011 Rosthern	0.5–2.5	PsC14542p181 - Sc5048_78539	5.15	0.15	14.53
LG7a	Yield (g/m^2^)	2012 Sutherland	2.7–5.1	Sc5048_78539 - Sc13836_25493	3.98	−36.70	12.53
LG7a	Seed starch conc. (%)	2012 Sutherland	5.2–8.0	Sc13836_25493 - PsC13063p113	6.60	−0.53	16.09
LG7a	Seed starch conc. (%)	2012 Rosthern	5.8–6.4	PsC17565p62 - PsC894p666	3.56	−0.28	7.58
LG7a	Seed starch conc. (%)	2010 Rosthern	5.9–6.6	PsC17565p62 - PsC6197p84	3.33	−0.34	10.82
LG7a	Seed zinc conc. (ppm)	2010 Rosthern	10.3–12.3	Sc1295_349656 - Sc3544_82453	3.67	0.75	6.34
LG7a	Yield (g/m^2^)	2011 Sutherland	15.6–16.8	Sc5607_100908 - PsC4676p597	3.10	−27.50	8.95
LG7a	Acid detergent fibre (%)	2012 Sutherland	17.2–20.5	Sc5607_100934 - Sc18622_41612	5.32	0.15	14.04
LG7a	Neutral detergent fibre (%)	2012 Sutherland	20.8–24.6	Sc18622_41612 - PsC5078p112	5.12	0.30	8.10
LG7b	Seed protein conc. (%)	2010 Sutherland	25.2–27.1	Sc20361_14817 - Sc3324_313865	3.31	−0.34	10.13
LG7b	Seed protein conc. (%)	2010 Rosthern	25.6–28.1	Sc20361_14817 - Sc3324_313865	3.88	−0.41	12.96
LG7b	Seed protein conc. (%)	2011 Sutherland	42.6–43.3	Sc8615_6507 - Sc986_91465	3.16	−0.32	9.82
LG7b	Seed protein conc. (%)	2012 Sutherland	86.5–87.0	PsC6994p243 - PsC6313p125	3.14	−0.31	9.28

Details of QTLs that were identified on the same linkage group in at least 50% of the trials were only listed

^a^Additive effect. Any positive value of additive effect indicates that the trait alleles are from Carerra and the negative additive effect indicates the trait alleles are from CDC Striker

^b^This flanking marker represents the centre of this 0.3 cM QTL region

^c^Mycosphaerella resistance is measured as area under disease progress curve (AUDPC)

**Table 7:** Details of quantitative trait loci identified for different traits in PR-15 population in repeated tests

Linkage group	Trait	Station/year	QTL interval (cM)	Flanking markers	LOD	Add. Effect^a^	% phenotypic variance explained
LG1b	Yield (g/m^2^)	2012 Sutherland	18.5–20.2	PsC10747p192 - Sc1080_21315	3.39	37.08	9.24
LG1b	Yield (g/m^2^)	2012 Rosthern	19.0–20.8	Sc8907_136614 - Sc4727_127502	3.16	19.16	6.71
LG3a	Yield (g/m^2^)	2012 Sutherland	0–1.0	PsC7190p130 - Sc6503_79334	5.45	−49.29	17.93
LG3a	Yield (g/m^2^)	2013 Rosthern	28.1–29.5	Sc11611_93482 - Sc1978_161130	3.11	40.03	10.19
LG3a	Yield (g/m^2^)	2012 Rosthern	58.1–63.8	Sc14164_1954 - Sc8290_63040	9.06		9.65
LG3a	Plant height (cm)	2013 Sutherland	98.5–115.3	Sc3030_71736 - Sc5836_42661	6.07	−2.34	16.65
LG3a	Plant height (cm)	2012 Rosthern	98.5–115.5	Sc3030_71736 - Sc5836_42661	12.99	−3.4	27.39
LG3a	Plant height (cm)	2013 Rosthern	98.8–115.4	Sc3030_71736 - Sc5836_42661	12.94	−3.86	33.3
LG3a	Lodging resistance (1–9)	2012 Sutherland	99.2–115.1	Sc3030_71736 - Sc5836_42661	9.15	0.43	31.3
LG3a	Days to maturity	2012 Rosthern	104.4–113.2	Sc3030_71736 - PsC17710p220	4.71	−0.97	15.04
LG3a	Lodging resistance (1–9)	2012 Rosthern	105.3–113.9	Sc3030_71736 - Sc5836_42661	8.83	0.61	28.5
LG3a	Yield (g/m^2^)	2013 Rosthern	120.4–127.9	Sc7171_14359 - Sc2661_275495	4.77	−49.04	15.09
LG3a	Days to maturity	2013 Rosthern	126.6 - 130.3	PsC13164p99 - Sc9107_65749	5.79	−0.57	18.00
LG3a	Days to flowering	2012 Rosthern	130.8–138.9	Sc9107_65749 - Sc9194_30175	4.69	−0.28	15.31
LG3a	Days to maturity	2012 Rosthern	139.4–142.0	Sc9194_30175 - PsC19244p283	3.33	−0.82	10.05
LG3a	Days to flowering	2013 Sutherland	140.3–142.7	Sc9194_30175 - Sc12608_67073	4.58	−0.36	11.63
LG3a	Yield (g/m^2^)	2013 Sutherland	140.7–141.0	PsC11929p181 - Sc1333_166281	4.67	−38.53	13.02
LG3a	Days to maturity	2012 Sutherland	141.0–143.1	PsC11929p181 - Sc12608_67073	3.51	−0.51	10.88
LG3a	Days to maturity	2013 Sutherland	141.5–144.4	Sc1333_166281 - Sc5805_247604	3.76	−0.33	7.98
LG3a	Plant height (cm)	2012 Sutherland	143.1–146.7	Sc12608_67073 - Sc6140_92759	4.35	−2.74	13.8
LG3a	Plant height (cm)	2013 Sutherland	148.6–152.7	Sc6140_92759 - PsC13040p226	3.47	−1.7	9.22
LG5	Days to maturity	2013 Sutherland	3.6–6.6	PsC20318p230 - Sc21433_13524	6.55	0.45	15.23
LG5	Days to flowering	2013 Sutherland	5.0–14.9	PsC713p412 - PsC26750p187	7.22	0.46	20.04
LG5	Days to flowering	2013 Rosthern	5.2–13.0	PsC713p412 - PsC26750p187	3.94	−0.34	14.89
LG5	Days to maturity	2013 Rosthern	6.0–11.5	PsC713p412 - PsC26750p187	3.64	0.44	11.41
LG5	Days to flowering	2012 Rosthern	91.6–94.1	Sc6512_5112 - PsC466p127	5.21	0.29	16.91
LG5	Seed phytate conc. (ppm)	2013 Rosthern	92.1–94.8	Sc12815_23289 - Sc4207_179095	6.69	−44.58	23.67
LG5	Seed phytate conc. (ppm)	2012 Rosthern	94.4–99.3	PsC466p127 - Sc15571_50927	12.48	−61.55	16.09
LG5	Days to maturity	2012 Rosthern	103.9–106.6	Sc1391_18953 - PsC11390p169	3.2	0.74	9.22
LG5	Seed phytate conc. (ppm)	2012 Sutherland	105.5–108.1	Sc19803_9214 - PsC18987p64	10.59	−60.15	31.32
LG5	Seed phytate conc. (ppm)	2013 Sutherland	105.5–108.1	Sc19803_9214 - PsC18987p64	11.37	−61.44	33.19
LG5	Days to maturity	2012 Sutherland	127.2–128.8	PsC1106p196 - Sc7339_115925	3.57	0.54	12.87

Details of QTLs that were consistent on the same linkage group in atleast two of the four trials were presented

^a^Additive effect. Any positive value of additive effect indicates that the trait alleles are from 1 to 2347-144 and the negative additive effect indicates the trait alleles are from CDC Meadow

##### QTLs for Mycosphaerella blight resistance

Mycosphaerella blight resistance was measured at four growth stages of PR-07 using a 0–9 rating scale and the scored values were used to calculate AUDPC. Of the QTLs identified on six linkage groups in six trials, the QTLs on LG3b were identified in four of the six trials (Table 3). These QTLs on LG3b shared overlapping linkage group positions in three trials, and the QTL representing this overlapping region had the maximum LOD of 8.9 and explained 26.3% of the phenotypic variance (Table 6).

##### QTLs for lodging resistance

All three mapping populations were evaluated for lodging resistance and significant QTLs were identified in all populations on linkage groups associated with chromosome 3. In all the populations, lodging scores measured at pod filling stage were used for QTL analysis. In PR-02, QTLs were identified on LG3b in two of the five trials (Table 2). In PR-07, QTLs on LG3b were identified in five of the six trials (Table 3). In PR-15, QTLs were identified on LG3a in two of the four trials while four other QTLs were located on different linkage groups in individual trials (Table 4). The major QTLs for lodging resistance identified on Chromosome 3 had a maximum LOD value of 8.8, 13.2 and 9.2 in PR-02, PR-07 and PR-15 populations, respectively, and these QTLs explained a phenotypic variance of up to 35.3% in PR-07 (Tables 2, 6 and 7).

##### QTLs for grain yield

Based on six trials, multiple QTLs were identified for grain yield in PR-02, PR-07 and PR-15. QTLs were mapped on LG6 of PR-02 in five of the six trials, LG3b of PR-07 in five of the six trials, and LG3a of PR-15 in all the four trials (Tables 2, 3 and 4). In PR-02, the QTL on LG6 was located in the same linkage group position in three of the five trials and this common QTL represented LOD values of 6.2 to 8.0 and explained phenotypic variance of 11.8 to 24.6% (Table 5). The QTL located on LG3b of PR-07 represented a maximum LOD value of 14.1 and explained 41.7% phenotypic variance. The same QTL explained a phenotypic variance of 22.6% in another trial (Table 6). The QTLs identified on LG3a of PR-15 were at different linkage group positions in all the four trials, and explained a phenotypic variance of up to 17.9%, while the QTLs identified on LG1b in two of the four trials shared the linkage group position and explained phenotypic variance of 6.7 to 9.2% (Table 7).

##### QTLs for seed weight

Seed weight of 1000 seeds was determined in two trials of PR-02 and QTLs have been identified on seven linkage groups, of which QTLs on LG3b and LG6 were identified in both trials (Table 2). QTLs on these two linkage groups represented a maximum LOD of 4.6 and explained a phenotypic variance of 11.6% (Table 5). For the same trait, multiple QTLs were identified on six linkage groups in PR-07, based on six trials. QTLs on three linkage groups LG3c, 4 and 6 were identified in at least four or more of the six trials tested (Table 3). The QTL on LG3c is the most significant QTL based on its overlapping linkage group positions in four of the five trials, with a maximum LOD of 15.6 and 40.0% phenotypic variance explained (Table 6).

#### QTLs for seed quality traits

##### QTLs for seed protein concentration

QTLs for seed protein concentration were mapped on LG1b and LG4a of PR-02 in two of the three trials tested (Table 2). In both the trials the identified QTLs were in the same linkage group positions. The QTLs on LG1b and LG4a represented a maximum LOD value of 5.0 and 3.4, and explained the phenotypic variance of up to 15.9 and 10.3%, respectively (Table 5). In PR-07, QTLs for seed protein concentration were identified on LG3b and LG7b in three and four of the six trials. These QTLs on LG3b and LG7b represented an average LOD of 3.7 and 3.4, and explained 11.7 and 10.6% phenotypic variance, respectively (Tables 3 and 6).

##### QTLs for acid detergent fibre (ADF)

In PR-02, based on three trials, QTLs were identified for ADF on four linkage groups, of which QTLs on LG7 were identified in two of the three trials (Table 2). The two QTLs on LG7 were in a common linkage group position with LOD values of 8.4 and 8.7 and explained a phenotypic variance of 28.0 and 26.2% (Table 5). In PR-07, QTLs were identified on seven linkage groups, of which QTLs on LG4 and LG7a were identified in four and three of the six trials, respectively. These QTLs which shared the same linkage group positions in two trials represented a maximum LOD of 6.6 and phenotypic variance of 18.7% (Table 6).

##### QTLs for neutral detergent fibre (NDF)

QTLs on multiple linkage groups were identified for NDF in PR-02, of which QTLs on LG5a were identified in all the three trials tested (Table 2). These three QTLs represented LOD values from 5.0 to 5.5 and phenotypic variance of 16.2 to 19.0% (Table 5). In PR-07, NDF QTLs were identified on LG1a in all the six trials and QTLs on LG4 and LG7a were identified in four and three of the six trials, respectively (Table 3). The QTLs on all these three linkage groups shared the same linkage group position in at least 50% of the trials and represented a maximum LOD of 19.5 and explained a phenotypic variance of up to 44.0% (Table 6).

##### QTLs for seed starch concentration

For seed starch concentration, multiple QTLs were identified on LG2b and LG4a of PR-02, and LG1a, LG3b, LG3c and LG7b of PR-07 (Tables 2 and 3). In PR-02, the QTLs on LG2b represented the same linkage group regions in two of the three trials tested with an LOD of 5.5 and 21.0% of phenotypic variance explained (Table 5). In PR-07, QTLs identified on LG1a, LG3b, LG3c and LG7a were significant in repeated trials, and the QTLs shared the linkage group positions in majority of the trials. The maximum LOD value represented by an individual QTL is 8.4 and this QTL on LG7a explained a phenotypic variance of 20.1% (Table 6).

##### QTLs for seed iron concentration

Based on two phenotypic trials, QTLs for seed iron concentration were identified on four linkage groups of PR-02, while the QTL on LG3b was highly significant in both the trials representing LOD values of 7.6 and 6.3 (Table 2). This QTL has the common linkage group position in both trials and represents a phenotypic variance of 20.7 and 26.7% (Table 5). In comparison, of the QTLs identified on six linkage groups of PR-07, the QTLs on LG3b were significant in all of the six trials tested (Table 3). QTLs on this linkage group shared the linkage group position in four of the six trials and represented a maximum LOD of 10.1 and phenotypic variance of 35.5% (Table 6).

##### QTLs for seed selenium concentration

QTLs were identified on three linkage groups of PR-02 based on two phenotypic trials, of which LG7 represented QTLs in both the trials within the same linkage group region and explained a phenotypic variance of up to 15.0% (Tables 2 and 5). In PR-7, QTLs were identified on LG4 and LG5b in two of the six trials (Table 3). The QTL on LG5b has the maximum LOD value of 4.5 and explained 17.1% of the phenotypic variance.

##### QTLs for seed zinc concentration

Four QTLs were identified one each on LG1a and LG3b, and two on LG6 of PR-02 in one of the two trials tested (Table 2). The QTL on LG3b was highly significant with an LOD value of 13.7 and 25.8% of phenotypic variance (Table 5). In comparison, the most significant QTLs for seed zinc concentration in PR-07 were also located on LG3b. One QTL on this linkage group positioned in the same region in three trials had an average LOD value of 15.5 and explained 42.7% of the phenotypic variance (Table 6). QTLs were also identified on LG7a in repeated trials with a maximum LOD of 5.9 and 11.7% of phenotypic variance explained (Table 6).

##### QTLs for seed dimpling

In PR-07, QTLs for seed dimpling were identified on four linkage groups with the QTL on LG5a being consistent in all six trials (Table 3). This QTL was mapped in the same linakge group position in all trials. The LOD and percent phenotypic variance explained by this QTL ranged from 4.1 to 10.4 and 12.7 to 29.2, respectively (Table 6).

##### QTLs for seed shape

In PR-07, 16 QTLs located on six linkage groups were identified for seed shape. The QTLs on LG3c were identified in all six trials, while the QTLs on LG7a were identified in four of the six trials (Table 3). The maximum LOD of QTLs on LG3c was 8.6, and the maximum phenotypic variance explained was 22.1%. The four QTLs identified on LG7a shared the linkage group position and the LOD and percent phenotypic variance explained ranged from 6.3 to 8.5, and 15.7 to 20.6, respectively (Table 6).

##### QTLs for seed phytate concentration

QTLs for seed phytate concentration were mapped in PR-15. QTLs for this trait were identified on three linkage groups, LG3a, LG5 and LG6a, of which the QTL on LG5 was identified in all four trials tested in two locations (Table 4). The four QTLs were positioned in two chromosome regions, with two trials from each location sharing the same region. The LOD values of these QTLs ranged from 6.7 to 12.5 and explained a phenotypic variance of 16.1 to 33.2% (Table 7).

## Discussion

Precise phenotyping is critical for accurate identification of marker trait association and presence of QTL [5]. This research was based on substantial sets of phenotypic data collected in three pea RIL populations. Phenotypic data arose from field trials which were managed under conditions typical of field pea production in western Canada. Field trials are superior to indoor trials in terms of their direct relevance to plant breeding and subsequent commercial applications. Agronomic traits (days to flower, days to maturity, plant height, lodging score, and grain yield) were assessed at four to six station/years, with two biological replications at each station/year. All field trials had low coefficient of variation for grain yield, and average to above-average mean grain yield indicating their reliability for use in QTL analyses. Relevant seed quality assessments (seed macro- and micro-nutrient concentration, seed weight, seed dimpling, and seed shape), including phytate concentration in the case of PR-15, were collected and in all cases useful variation was noted. The most significant trait associations observed among the three RIL populations were between days to flowering and days to maturity, days to maturity and yield, and Mycosphaerella blight severity and lodging.

In this research, we identified QTLs for many important traits for pea breeding, based on extensive phenotyping in field trials and high density genetic linkage maps based on GBS and Illumina GoldenGate array genotyping. Some of the traits evaluated in this study are highly quantitative in nature, and are important for pea breeding. Some of the QTLs identified for individual traits were also compared between the mapping populations.

We used the GBS protocol as described by Elshire et al. [13] to genotype the pea mapping populations because of its potential for SNP discovery across the entire genome and the availability of a reference draft genome provided by the International Pea Genome Consortium to facilitate read alignment and SNP calling. GBS has been successfully used in many different crops to score co-dominant SNP markers across the genome and to generate high density genetic linkage maps [35]. Based on whole genome sequencing of four pea lines, Boutet et al. [19] identified 419,024 SNPs and a subset of 64,754 SNPs were genotyped by sequencing of RILs of the cross Baccara/PI180693, thus indicating the potential of sequence based genotyping methods for simultaneous genotyping of a number of SNPs in pea. In our study, GBS was successful in identification of > 25,000 SNPs in each mapping population, and after filtering 2234, 3389 and 3541 SNP markers were used for genetic linkage map construction in PR-02, PR-07 and PR-15 mapping populations, respectively. In a similar study, using *Ape*kI restriction digestion based GBS, Ma et al. [36] identified 1609 high quality SNPs in a pea RIL population derived from Aragorn x Kiflica. To improve the quality of the genetic linkage maps, all SNP markers with > 15% missing data were omitted from map construction, though some important SNPs might have been eliminated. Liu et al. [37] suggested utilization of SNPs with < 20% missing data without imputation to improve the data quality of GBS genotyping. The SNP markers genotyped by GBS were anchored with the existing SNP markers genotyped in the same populations [10] for assignment of linkage groups as well as generation of dense linkage maps. The average distance between the mapped loci was 1.57 cM, and the identified GBS markers were distributed across the seven chromosomes of pea. The dense genetic linkage maps developed in the current study will facilitate further fine-mapping of the identified QTLs for agronomic and seed nutritional traits.

The genetic linkage maps developed in this study were used to identify QTLs for multiple traits in multiple environments. The phenotypic data from each station/year were used independently for identification of QTLs and their consistency across environments. In interpreting the results for identification of reliable trait-linked markers, QTLs which were identified on the same linkage groups in repeated trials were considered, and the next level of comparison was made through comparison of QTL positions in repeated trials. Overall, 375 QTLs were identified for various traits including days to flowering, days to maturity, plant height, Mycosphaerella blight resistance, lodging resistance, seed mineral concentration, starch and fibre concentration, seed weight and grain yield, known to be a highly complex trait. Highly significant and reproducible QTLs were identified for plant height, lodging resistance, yield and seed iron concentration in more than one population.

Although flowering time is a quantitative trait controlled by multiple genes, in pea *HIGH RESPONSE TO PHOTOPERIOD* (*HR)*, an ortholog of *EARLY FLOWERING* 3 (*ELF3*), has been identified as a gene involved in Circadian clock function, which controls a significant proportion of flowering time variation in global pea germplasm [38]. Henaut et al. [39] confirmed the association of the *HR* locus with QTL for flowering time and the co-localization of this locus with a major QTL affecting winter frost tolerance. Vanhala et al. [40] reported that in the absence of HR, flowering time in a collection of pea accessions was affected by the LATE FLOWERING (LF) gene, and positively correlated with the length of the growing season in the region of origin of the accession. In the current study, the major QTL for flowering time was localized on LG6 in PR-02. This QTL was identified in five of the six field trials tested, shared overlapping linkage group positions, and explained phenotypic variance of up to 47.9%. Based on the known linkage between *HR* locus and flowering time response in pea from previous studies, this QTL on LG6 could be the *HR* locus. Identification of QTLs for flowering time in PR-07 and PR-15 on different linkage groups could be because of the absence of *HR* locus in these populations.

Highly significant QTLs for plant height were identified in all three mapping populations. One QTL each on LG3b of PR-02, LG3c of PR-07, and LG3a of PR-15 identified in repeated trials, contributed to a phenotypic variance of 41.5, 65.1 and 33.3% in the three populations, respectively. Tar’an et al. [22] reported three QTLs related to plant height in a RIL population tested in 11 environments, and the QTLs together explained 65% of the phenotypic variance. Hamon et al. [41] identified three minor QTLs for plant height in another RIL population and these QTLs were linked to *Aphanomyces* root rot resistance. Plant height is known to be associated with other traits such as lodging resistance and Mycosphaerella blight resistance. The QTLs identified for plant height in the current study are well suited for MAS in breeding programs as they explain 33–65% of the phenotypic variance. Similar to the observations in pea, plant height in chickpea was identified as a quantitative trait governed by six QTLs [42].

In pea, improvement of lodging resistance is an important breeding objective to improve air circulation in the canopy as a means of reducing fungal disease development, and to facilitate harvest. Only a few studies have reported QTLs associated with this complex quantitative trait, which is also highly dependent on environmental factors. Tar’an et al. [22] reported two QTLs for lodging resistance which explained 58% of the phenotypic variance in 11 environments tested. In the current study, we identified a major QTL for lodging resistance on linkage group 3c of PR-07 in the majority of the environments tested and this QTL explained a phenotypic variance of up to 35.3%. In PR-15 the major QTL was located on LG3a and shared the linkage group region in two of the four environments tested and explained a phenotypic variance of 31.3 and 28.5%. A major QTL for lodging resistance in a pea RIL population derived from Delta x RER which explained 49.9% of the phenotypic variance was also located on linkage group 3 [43]. The major QTLs for lodging resistance in both PR-02 and PR-07 overlapped with the QTLs for plant height identified in these populations. Tar’an et al. [22] also reported that QTLs associated with lodging susceptibility in pea were also associated with plant height.

Mycosphaerella blight resistance is another complex quantitative trait important for pea breeding. Lodging susceptibility is known to increase the severity of Mycosphaerella blight [44] and Mycosphaerella resistance QTLs are known to be associated with lodging resistance [17]. We have identified QTLs for Mycosphaerella resistance on LG3b and LG4 in PR-07. These QTLs explained phenotypic variation of 10.5–26.3%. The position of the QTLs within these linkage groups varied from one trial to another with the exception of one QTL on LG4 identified in two trials, thus indicating the effect of environment on disease resistance. Using an interspecific pea RIL population, Jha et al. [30] identified QTLs for Mycosphaerella resistance on LGIII and LGIV in repeated field trials. Most of the Mycosphaerella resistance QTLs identified in other mapping populations were also located on LGIII [45, 46].

Grain yield is a complex quantitative trait and we have identified multiple QTLs for grain yield in different mapping populations. The QTLs on LG4a and LG6 in PR-02, LG3c and LG7b of PR-07, and LG1b of PR-15 were identified in repeated trials and explained a phenotypic variance of 6.7 to 54.2%. The QTL on LG3c of PR-07 is of particular importance as it was identified in five of the six trials and explained a phenotypic variance of 13.3 to 21.6%. Several QTLs for grain yield have been reported earlier. Tar’an et al. [47] identified QTLs for grain yield which together explained 39% of the phenotypic variance and the major QTLs were located on LGII. Krajewski et al. [48] identified multiple QTLs for grain yield in pea. In their study, the QTLs were identified on different linkage groups, while the major QTLs were located on LG5, with minor QTLs on LG2. Since grain yield is affected by genotype x environment interactions at both vegetative and reproductive growth stages, variation in the QTLs identified in different mapping populations and different environments is expected. Annicchiarico et al. [49] reported co-localization of SNP markers associated with early flowering and high yield of pea in a genome-wide association study (GWAS). In the current study, we have noted co-localization of QTLs associated with days to flowering and grain yield on LG6 of PR-02, LG3b of PR-07 and LG3a of PR-15.

The effect of days to flowering loci on other traits including yield was determined by using the days to flowering loci as control markers in cofactor analysis. The cofactor analysis altered the LOD scores of QTLs for other traits on the same linkage group from 0 to 35%. For example, in the PR-07 trial at Sutherland in 2011, using days to flowering loci on LG3b as control markers did not alter the LOD score of Mycosphaerella blight severity QTL on the same linkage group, increased the LOD of plant height QTL from 11.90 to 17.00, and decreased the LOD of other QTLs including yield from 3.75 to 3.51, 1000 seed weight from 4.97 to 3.59, ADF from 10.1 to 6.65, NDF from 7.44 to 5.33, seed zinc concentration from 6.61 to 4.6.

QTLs for other traits including fiber and starch concentration, seed mineral concentration, seed phytate concentration of mature pea seeds were also identified in this study. Some of these QTLs were highly significant and identified in repeated trials, and thus have potential for MAS in pea breeding. For example, the QTL for seed zinc concentration identified on LG3c of PR-07 was identified in four of the six trials and explained a phenotypic variance of 12.3 to 43.2%. Ma et al. [36] reported that the QTL with highest explanation of phenotypic variance for seed zinc concentration in a mapping population derived from Aragorn x Kiflica tested in two different locations was also located on LG3. Shunmugam et al. [50] also reported a major QTL for seed phytate concentration in pea on LG5, and this QTL was confirmed here using an expanded set of markers.

## Conclusions

The current study has identified many QTLs for quantitative traits in pea using GBS resources and dense genetic linkage maps. QTLs for flowering time, plant height and lodging resistance were identified consistently in field trials. There is a potential for fine mapping these QTLs, identification of trait-related genes using the current QTL information, and for using the marker information for MAS in breeding programs. The highly significant and reproducible QTLs identified for days to flowering (PV_max_ = 47.9%), plant height (PV_max_ = 65.1%), lodging resistance (PV_max_ = 35.3%), grain yield (PV_max_ = 54.2%), seed iron concentration (PV_max_ = 27.4%), and seed zinc concentration (PV_max_ = 43.2%) can be used for immediate breeding applications. We are in the process of converting the SNP markers flanking the identified QTL regions for these traits into simple assays, and validating the markers using a genome wide association study (GWAS) panel of 175 genotypes which were phenotyped in multi-location, multi-year replicated field trials.

## Additional files

Additional file 1: Minimum, maximum and mean values of phenotypic traits measured in the parents (Orb and CDC Striker) and the PR-02 population. (XLS 14 kb)

Additional file 2: Minimum, maximum and mean values of phenotypic traits measured in the parents (Carrera and CDC Striker) and the PR-07 population. (XLS 19 kb)

Additional file 3: Minimum, maximum and mean values of phenotypic traits measured in the parents (1–2347-144 and CDC Meadow) and the PR-15 population. (XLS 10 kb)

Additional file 4: Combined correlation eoefficients between phenotypes measured in PR-02 RIL population in six trials. (XLS 7 kb)

Additional file 5: Combined correlation eoefficients between phenotypes measured in PR-07 RIL population. (XLS 15 kb)

Additional file 6: Combined correlation eoefficients between phenotypes measured in PR-15 RIL populations. (XLS 10 kb)

## Abbreviations

DTF: Days to Flowering
DTM: Days to Maturity
GBS: Genotyping-By-Sequencing
GWAS: Genome-wide Association Study
MAS: Marker Assisted Selection
NGS: Next Generation Sequencing
QTL: Quantitative Trait Loci
SNP: Single Nucleotide Polymorphism
SSR: Simple Sequence Repeats
